# Occupational Therapy Referral Pathways for Upper Limb Conditions in Tygerberg, South Africa: Stakeholder Perspectives

**DOI:** 10.1155/oti/5528214

**Published:** 2026-10-05

**Authors:** Truwin Appel, Hilletje de Villiers, Buhlebethu Fihlani, Cherise Reyneke, Khanyisa Tshalala, Susan de Klerk

**Affiliations:** ^1^ Division of Occupational Therapy Faculty of Medicine and Health Science Stellenbosch University Stellenbosch South Africa sun.ac.za; ^2^ Undergraduate Occupational Therapy Student Division of Occupational Therapy Faculty of Medicine and Health Science Stellenbosch University Stellenbosch South Africa sun.ac.za

## Abstract

Effective referral pathways to occupational therapy are essential for continuity of care and recovery following upper limb injury or condition. Although challenges within South Africa′s public healthcare referral systems are recognised, patients′ experiences remain underexplored. This study explored patients′ experiences of occupational therapy referral pathways within the Tygerberg substructure and identified factors influencing access to care. A qualitative descriptive design was used, with semistructured interviews conducted with seven purposively sampled participants. Data were analysed thematically using Braun and Clarke′s framework. Three themes emerged: challenges in healthcare accessibility and referral processes; environmental, administrative and communication barriers affecting access to occupational therapy; and factors facilitating access and positive rehabilitation experiences. Findings highlighted the influence of health literacy, communication, transport, distance, community safety and referral delays on access to services. Despite these barriers, participants reported positive perceptions of occupational therapy and its outcomes. The findings emphasise the need for patient‐centred referral pathways, improved patient education and enhanced communication within public healthcare rehabilitation services.

## 1. Introduction

Optimal upper limb function is essential for participation in daily activities and engagement with the environment. Upper limb injuries and conditions can disrupt this participation and affect an individual′s independence, efficiency and ability to complete tasks. De Klerk et al. [1] found that upper limb injuries influence the time required to perform activities, levels of independence and attitudes towards task performance. Following an upper limb injury or condition, occupational therapy intervention may be required to support recovery and functional participation. Patients who attend follow‐up rehabilitation demonstrate greater community involvement and participation in daily activities than those who do not. Van Stormbroek et al. [2] further emphasise the importance of rehabilitation across the lifespan, particularly for individuals with upper limb injuries, as impairment of the upper limb may negatively affect employment and livelihood.

Despite the importance of rehabilitation, South African studies have identified significant barriers to accessing these services. These include the cost of care, limited transport, geographical challenges, resource constraints, poor attitudes among rehabilitation staff and ineffective referral pathways [2–6]. Access to occupational therapy is therefore influenced not only by service availability but also by the effectiveness of healthcare referral systems.

South Africa′s public healthcare system operates through a hierarchical referral structure comprising primary healthcare (PHC) clinics, community health centres, district hospitals, regional hospitals and tertiary hospitals. Patients are expected to enter the system at PHC level and be referred according to their healthcare needs. However, patients frequently bypass lower levels of care because of perceived inefficiencies, resulting in increased pressure on higher‐level facilities. Within the Tygerberg substructure, the healthcare network includes PHC clinics, community day centres, secondary hospitals and Tygerberg Hospital, a major tertiary referral centre in the Western Cape. Referral pathways may therefore involve movement between clinics, hospitals and community‐based services depending on patient needs and service availability.

Several challenges within South African referral systems have been identified. According to the Referral Policy of the South African National Health Service [7], these include poor compliance with referral policies, communication gaps between healthcare providers and shortages of transport and human resources. In addition, relationships between community and home‐based care providers and public healthcare facilities are often poorly defined, contributing to high rates of self‐referral to hospitals. These challenges may result in fragmented care, reduced continuity of services and decreased retention within the healthcare system. Consequently, the effectiveness of healthcare delivery is closely linked to the efficiency of referral pathways.

Mirt et al. [8] highlight a gap in research examining nonsurgical treatment pathways from patient and healthcare provider perspectives, particularly within healthcare systems facing resource and organisational challenges. Within the South African context, and specifically the Tygerberg substructure, little is known about patients′ experiences of occupational therapy referral pathways following upper limb injury or condition. Furthermore, the challenges encountered during movement between levels of care have not been adequately explored. Understanding these experiences is important, as barriers within referral pathways may influence attendance at therapy, adherence to treatment and ultimately rehabilitation outcomes. These outcomes extend across all domains of the International Classification of Functioning, Disability and Health (ICF) [9]. The ICF provides a framework for understanding how health conditions interact with contextual factors to influence body functions, activities and participation. Delays, poor communication and restricted access to services may therefore negatively affect physical recovery, performance of daily activities and participation in social and occupational roles.

Given the influence of referral systems on healthcare outcomes, understanding patients′ experiences of these pathways is essential. As the primary users of healthcare services, patients can provide valuable insight into factors that facilitate or hinder access to occupational therapy. Such knowledge may support the development of more efficient, client‐centred and responsive healthcare services that address the needs of patients and their communities [10]

Therefore, this study is aimed at exploring and describing patients′ experiences of occupational therapy referral pathways within the Tygerberg substructure following upper limb injuries or conditions. The study further sought to identify and analyse the challenges and opportunities within these referral pathways and their potential influence on access to occupational therapy services.

## 2. Method

### 2.1. Research Design

This study formed part of a larger three‐component research project conducted within Tygerberg Hospital and its substructures that explored referral processes from the perspectives of occupational therapists, doctors and patients. The findings of the first two components will be reported elsewhere. The current study adopted an interpretivist ontology, phenomenological tradition, qualitative paradigm and descriptive design to explore the experiences of patients with upper limb injuries or conditions referred within the Tygerberg substructure. Semistructured interviews and journey mapping were used to capture participants′ experiences and perspectives of the referral pathway [11, 12]. Findings were interpreted in relation to participants′ individual experiences. Ethical approval was obtained from Stellenbosch University′s Undergraduate Research Ethics Committee (HREC Reference No: U24/11/363).

### 2.2. Study Population

The study population comprised adults aged 18 years and older residing in the Western Cape who had sustained an upper limb injury or condition and had been referred to occupational therapy within the Tygerberg substructure. Referrals could occur between different levels of care, including primary, secondary and tertiary services. Occupational therapy services within the Tygerberg substructure are provided at PHC facilities, community health centres, district hospitals and Tygerberg Hospital, a tertiary referral hospital. Participants in this study were recruited from occupational therapy services at PHC facilities and Tygerberg Hospital. Patients are referred between levels of care by healthcare professionals according to clinical need. Referrals are predominantly paper based, with referral information documented in a referral letter that accompanies the patient to the receiving facility. The referral letter is typically placed in a sealed envelope and transported by the patient to support continuity of care and communication between facilities. Participants were required to speak isiXhosa, English or Afrikaans, the three most commonly spoken languages in the Western Cape. Purposive sampling was used to recruit seven participants who met the selection criteria (Table 1), allowing for an in‐depth exploration of their experiences.

**Table 1:** Selection criteria.

Selection criteria	Motivation
Participants aged 18 years and older.	Adults were selected because they are able to provide informed consent independently, without requiring parental or guardian consent. This enabled the collection of participants′ own experiences and perspectives regarding the referral process.
Participants with an upper limb injury or condition who had not yet been discharged from occupational therapy services.	These participants were able to provide current and relevant accounts of their experiences of the occupational therapy referral pathway, including barriers and facilitators encountered while accessing treatment.
Participants whose home language was English, Afrikaans or isiXhosa.	Inclusion of the three most commonly spoken languages in the Western Cape increased the potential participant pool. The research team was proficient in these languages, facilitating effective communication, transcription and data analysis.
Individuals with a previous traumatic brain injury or intellectual disability were excluded.	Participants were required to understand interview questions and communicate their experiences clearly to ensure the collection of rich and accurate data for thematic analysis.

### 2.3. The Researchers

The research team consisted of five final‐year (fourth year) occupational therapy students at Stellenbosch University, supervised by a senior lecturer from the Division of Occupational Therapy. The researchers represented diverse cultural and linguistic backgrounds, enabling interviews to be conducted in participants′ preferred languages. Each interview was conducted by two researchers, with at least one fluent in the participant′s preferred language. Regular team discussions were used to minimise individual bias and support reflexive interpretation of the data.

### 2.4. Context

Data were collected at two primary‐level healthcare facilities and Tygerberg Tertiary Hospital within the Tygerberg substructure that serve communities from predominantly lower socioeconomic backgrounds. Participants were recruited from occupational therapy services at these facilities.

### 2.5. Data Collection

A gatekeeper, who was an occupational therapist at each facility, received information about the study and participant selection criteria. Eligible patients were identified by the gatekeepers and invited to participate. Written informed consent was obtained before data collection. Eight face‐to‐face semistructured interviews and journey mapping activities were conducted. A semistructured interview guide was developed and standardised in English, Afrikaans and isiXhosa to ensure consistency across interviews and languages. Interviews were conducted in participants′ preferred languages by bilingual members of the research team, audio‐recorded, transcribed verbatim and translated into English where required. Translations were reviewed by a second researcher to ensure accuracy and preserve meaning. Recordings were stored securely, and pseudonyms were assigned to protect confidentiality.

### 2.6. Data Analysis

Data were analysed using Braun and Clarke [13] thematic analysis framework. Following transcription, researchers familiarised themselves with the data through repeated reading of the transcripts and identification of preliminary codes. Through iterative discussion and analysis, 31 codes were generated and grouped into nine categories, which subsequently informed three overarching themes. An inductive thematic approach was used to identify challenges and opportunities within occupational therapy referral pathways. ATLAS.ti assisted with data organisation, coding and theme development. Credibility and confirmability were enhanced through triangulation, audit trails and peer debriefing.

## 3. Findings

The participants consisted of three males and five females aged 24–69 years. Participants presented with upper limb conditions resulting from trauma, repetitive strain injuries and arthritic conditions. Interviews were conducted between March and May 2025 at three facilities within the Tygerberg substructure. Although eight participants were interviewed, only seven transcripts were included in the analysis due to technical difficulties during the recording of Interview 3. Participant 3 retained their identifying number for consistency but was excluded from data analysis. The themes, categories and subcategories that emerged from the analysis are presented in Table 2.

**Table 2:** Coding framework.

Themes	Categories	Subcategories
Challenges in healthcare, accessibility and occupational therapy referral pathway (Barriers and difficulties experienced by patients with upper limb injuries or conditions when accessing healthcare services and traversing the occupational therapy referral pathway within the Tygerberg substructure)	Gaps in service delivery and professionalism	● Attitude and ethics of staff
		● Patient concerns overlooked
		● Emergency transport gaps
	Limited patient knowledge of healthcare services, systems and staff	● Confusion about healthcare systems and services
		● Exposure‐informed knowledge of occupational therapy
		● Inadequate knowledge of occupational therapy services
		● Patients′ understanding of the general referral pathway
		● Lack of engagement with referral letter
	Referral methods to occupational therapy	● Paper‐based referral to occupational therapy
		● Patient verbal referral to occupational therapy
Patients′ perspectives of compounded barriers undermining reasonable access to occupational therapy (Environmental challenges, time‐related administrative delays and limited healthcare provider communications create barriers that influence participant′s reception of occupational therapy services and ability to access these services at referred facilities)	Contextual and environmental barriers to access occupational therapy services	● Barriers to public transport use and access
		● Distance from healthcare facilities
		● Environmental barriers to attendance
		● Violence in communities as a barrier to accessing healthcare facilities
	Inadequate communication about occupational therapy services	● Lack of comprehension due to complex language use
		● Limited provider communication on OT referral and services
	Time‐related administrative and referral delays	● Long waiting time for referral to occupational therapy
		● Prolonged wait for occupational therapy appointments
		● Waiting time due to paper filing system
Factors contributing to effective occupational therapy access and positive patient perceptions of outcomes and services. (Patient perceptions of positive experiences in the referral pathway to occupational therapy)	Successful appointment and occupational therapy input outcomes	● Appointment prioritisation
		● Minimal delay postoccupational therapy referral
		● Successful occupational therapy outcome following referral
	Ease of access and attendance at referred facility	● Attended occupational therapy at referral facility
		● Clarity on referral area navigation
		● Facility near patients
		● Patient preferred facility
	Stakeholder positive perception and satisfaction with overall referral and rehabilitation services	● Referral purpose to occupational therapy communicated
		● Perceived provider collaboration in referral
		● Positive occupational therapy referral experience
		● Positive perception of referral to occupational therapy
		● Positive perception of occupational therapy expertise

### 3.1. Theme 1: Challenges in Healthcare, Accessibility and Occupational Therapy Referral Pathway

This theme describes the barriers experienced by patients with upper limb injuries or conditions when accessing healthcare services and navigating the occupational therapy referral pathway within the Tygerberg substructure. It includes gaps in service delivery, limited understanding of healthcare services and professional roles and variations in referral methods.

#### 3.1.1. Gaps in Service Delivery and Professionalism

These experiences were relevant to occupational therapy referral pathways because they influenced participants′ entry into the healthcare system, progression between levels of care and access to subsequent occupational therapy services. Delays and negative interactions with healthcare professionals therefore affected not only initial treatment experiences but also the broader referral journey. Participants reported several challenges related to healthcare service delivery. Limited access to timely ambulance services emerged as a significant barrier. Participant 1 was unable to access an ambulance and therefore arranged personal transport to reach emergency care. Similarly, Participant 2 waited approximately 2 h for an ambulance after initially requesting assistance, delaying both treatment and the referral process. These experiences influenced access to subsequent healthcare services, including occupational therapy, by delaying entry into care and shaping participants′ engagement with the broader referral process. Concerns regarding professional attitudes and conduct were also reported. Participant 1 described an experience in which healthcare staff appeared unwilling to provide assistance or reassurance, contributing to emotional distress during treatment. This was reflected in the statement: “I think they (staff at the healthcare facility) were causing me to panic.” Participant 6 similarly suggested that receiving favourable treatment depended on maintaining a positive attitude towards staff: “Yes they…you must be nice to them, and they are nice to you.” Participants also described instances where healthcare providers dismissed their concerns. Participants 6 and 8 felt that reports of pain were minimised and considered insufficient to justify further assessment or referral. Participant 8 commented: “I think that people that work at triage should help people, even if the pain is not that bad.”

#### 3.1.2. Limited Patient Knowledge of Healthcare Services, Systems and Staff

Participants demonstrated limited understanding of healthcare services, professional roles and referral pathways. This lack of knowledge appeared to affect their ability to navigate the healthcare system effectively. Participant 1 indicated uncertainty regarding the distinction between clinics and hospitals. Similarly, participants were often unclear about the roles of different healthcare professionals. Participants 4 and 7 associated occupational therapy primarily with physiotherapy and described occupational therapists′ roles mainly in relation to healing, exercises and joint mobility. Limited engagement with referral letters further contributed to this lack of understanding. Of the seven participants, only Participant 2 reported reading the referral letter. The remaining participants either did not know a referral letter existed, did not know where it was located or were unsure whether they were permitted to read it. Only Participant 7 was aware of the letter but chose not to read it. Most participants demonstrated a basic understanding of referral processes, describing referral as being directed from one person or service to another to obtain appropriate care. However, Participants 2 and 6 showed limited understanding of the concept of referral itself. A lack of awareness regarding occupational therapy was evident across all participants. None reported knowing what occupational therapy involved before receiving treatment. Participant 2 stated: “I did not know what therapy means, so I only knew after I was here already.” The participant further indicated that additional information regarding recovery and referral processes would have been beneficial.

#### 3.1.3. Referral Methods to Occupational Therapy

Analysis indicated that occupational therapy referrals within the Tygerberg substructure relied on a combination of paper‐based and verbal communication methods. These methods appeared to influence patient engagement with both the referral and the broader referral process. Paper‐based referrals were the most common method. Six participants reported receiving either a written note in their patient file or an instruction on an appointment card to present at their occupational therapy appointment. However, only Participants 2 and 7 were aware that a formal referral letter existed. This suggests that paper‐based documentation was frequently used during the referral process, although patients were often unaware of its purpose or content. Paper‐based referrals were supplemented by verbal communication. All participants reported receiving verbal instructions from healthcare providers regarding their referral. These instructions generally consisted of brief directions explaining where to attend for occupational therapy services. Participant 5 described the referral process as: “word of mouth, but with my file.”

### 3.2. Theme 2: Patients′ Perspectives of Compounded Barriers Undermining Reasonable Access to Occupational Therapy

This theme describes how environmental challenges, communication barriers and administrative delays influenced participants′ ability to access occupational therapy services and shaped their experiences of the referral process.

#### 3.2.1. Contextual and Environmental Barriers to Access Occupational Therapy Services

Participants identified several environmental factors that made it difficult to attend occupational therapy appointments. Transport challenges were commonly reported by Participants 1, 2 and 4. These included limited availability of public transport, overcrowding of vehicles and the need to use multiple taxis to reach the referred facility. Distance to healthcare facilities further compounded these challenges. Participant 2 reported needing to use more than three minibus taxis to travel to the referred facility. For individuals who walked to appointments, long distances created an additional barrier to maintaining continuity of care. Weather conditions also affected access to services. Participants 6 and 8 described rain and cold weather as factors that restricted mobility and reduced their ability to attend appointments. Violence and crime within communities emerged as another barrier. Participants 4, 6 and 8 reported concerns about personal safety when travelling, particularly during adverse weather conditions. Participant 6 explained: “I can walk here. If it′s raining maybe I can take a taxi. I can maybe take an Uber. But the problem these days, now they rob the Ubers, the Ubers don′t want to come into your area.”

#### 3.2.2. Inadequate Communication About Occupational Therapy Services

Participants reported receiving limited information about occupational therapy and the reasons for referral. This lack of communication was often compounded by the use of medical terminology or complex language that was difficult to understand. As a result, participants had limited awareness of the purpose and potential benefits of occupational therapy services. Five of the seven participants stated that the referring healthcare professional did not explain why they were being referred to occupational therapy. Instead, they were simply instructed to attend the rehabilitation department. Participant 5 also indicated that the language used by healthcare providers sometimes made information difficult to understand. Participant 8 summarised this experience: “They did not explain to me as to why I needed to see an occupational therapist, so a bit more information there would have been appreciated.”

#### 3.2.3. Time‐Related Administrative and Referral Delays

Participants frequently reported delays in accessing occupational therapy services. These delays were attributed to slow referral processes, long waiting periods for appointments and administrative challenges associated with paper‐based filing systems. Participants 1 and 2 described lengthy delays between injury, referral and their first occupational therapy appointment. Participant 2, who sustained a stab wound to the volar aspect of the forearm, reported waiting 2 weeks after surgery before attending an occupational therapy session. The participant also expressed concern about the lengthy wait for appointments and the possibility of extended delays if an appointment was missed. Participant 4 reported a similar experience: “It was a long time, yes. The date that they gave me. Because I also thought to myself, but it is quite long.” Participant 4 was referred to occupational therapy in January, received an appointment date in February and attended the first session in March. Administrative difficulties also affected access to care. All three healthcare facilities used paper‐based folders and files to store patient information, which occupational therapists were required to access before appointments. Participants 4 and 8 reported delays when their files were misplaced or already in use, resulting in longer waiting times before treatment could commence. Overall, participants perceived administrative inefficiencies and referral delays as significant barriers to timely access to occupational therapy services.

### 3.3. Theme 3: Factors Contributing to Effective Occupational Therapy Access and Positive Patient Perceptions of Outcomes and Services

In contrast to Themes 1 and 2, this theme highlights participants′ positive experiences of the occupational therapy referral pathway. Participants described timely access to services, successful rehabilitation outcomes, convenient attendance at referred facilities and overall satisfaction with occupational therapy services.

#### 3.3.1. Successful Appointment and Occupational Therapy Input Outcomes

Although some participants experienced referral delays, Participants 6 and 7 reported receiving occupational therapy appointments soon after referral. Prompt scheduling was viewed positively and contributed to favourable experiences of the referral process. Attendance commitment emerged as a common factor among participants. Five participants emphasised the importance of prioritising appointments despite personal or environmental challenges. Participant 4 expressed determination to attend all appointments, whereas Participant 5 reported making every effort to attend on the scheduled date. Participant 7 stated that leave was taken from work to ensure attendance at therapy sessions. Similarly, Participant 8 attended therapy despite negative community perceptions of occupational therapy services. Participants 5, 6 and 8 identified the desire to improve their health as the primary motivation for attending appointments. Participant 6 explained: “I make preparations, and I make time…I get up earlier, because that′s my time since then I know at the end of the day, I know what′s going on.” Participants also reported positive outcomes following occupational therapy intervention. Participants 1, 2, 4, 5 and 8 described improvements in their upper limb conditions after receiving therapy. Reported benefits included improved function, reduced pain, increased knowledge of assistive technology and a better understanding of precautions related to their condition.

#### 3.3.2. Ease of Access and Attendance at Referred Facility

All participants reported attending occupational therapy services at the facilities to which they had been referred. Convenient access to these facilities contributed to positive attendance experiences and overall satisfaction. Participants 5 and 6 explicitly stated that they attended therapy at their referred facilities. Although Participants 1, 2, 4, 7 and 8 did not directly state this, their journey maps indicated attendance at the facilities to which they had been referred. Proximity to home emerged as a key factor influencing facility preference. Participants 4, 5 and 6 reported that the short travel distance made attending appointments convenient. Participants 4, 5, 6 and 8 indicated that they could walk to the facility, further reducing barriers to attendance. Participant 6 stated: “This is the best for me here, rather than traveling far…I can walk here.” In addition to proximity, familiarity with the area and healthcare facility influenced participant preferences. Participant 7 reported preferring the referred facility because it was both convenient and familiar. Clear directions from healthcare providers further supported attendance. Participants 1, 2, 5, 6, 7 and 8 reported receiving instructions that enabled them to locate occupational therapy services easily. Participant 1 noted: “They did tell me which floor it was and all of that.”

#### 3.3.3. Stakeholder Positive Perception and Satisfaction With Overall Referral and Rehabilitation Services

Participants′ positive perceptions of occupational therapy services were influenced by clear communication, collaborative healthcare practices, professional expertise and successful rehabilitation outcomes. Participants 1 and 7 reported that the purpose of the occupational therapy referral was clearly explained by the referring healthcare professional. This understanding appeared to contribute positively to their experience of the referral process. Participants 1, 2, 7 and 8 described collaboration among healthcare professionals involved in their care. Participant 1 specifically stated that coordinated healthcare services contributed to recovery and supported progress throughout rehabilitation. Most participants believed that referral to occupational therapy was necessary and beneficial. Six of the seven participants felt that occupational therapy played an important role in their recovery. Participant 2 remarked that referral to therapy was a good recommendation by the doctors. Participant 8 similarly stated: “After all the help and education I received, I do think that it was necessary for me to come here yes.” Many participants described the referral and rehabilitation process positively. Participants 1, 4, 5 and 7 specifically highlighted aspects of care that supported their recovery. Reported benefits included improvements in body functions, such as reduced pain and enhanced upper limb function; improved activity performance in everyday tasks; and increased knowledge of assistive technology and condition‐specific precautions, which supported self‐management and participation in daily life. Participants also expressed appreciation for the occupational therapists′ interpersonal skills and professional conduct. Participant 4 described the therapist as empathetic, supportive and focused on the patient′s best interests: “The therapist is a people′s person. He treats his patients well. He talks to you so that you can understand. He says that at the end of the day it is for your best will.” Participant 5 expressed similar views regarding the quality of care received. In addition, five participants reported satisfaction with the knowledge and competence demonstrated by occupational therapists. Professional expertise was therefore an important contributor to positive rehabilitation experiences and overall satisfaction with services. In addition, five participants reported satisfaction with the knowledge and competence demonstrated by occupational therapists.

## 4. Discussion

This study explored participants′ perspectives of the occupational therapy referral process and identified challenges and opportunities experienced by individuals with upper limb injuries or conditions within the Tygerberg substructure. The findings suggest that access to occupational therapy is shaped by a combination of patient‐related, environmental and health system factors. Although several barriers limited engagement with referral pathways, participants also identified factors that facilitated access and positively influenced rehabilitation experiences.

### 4.1. Theme 1: Healthcare Navigation and Understanding of the Referral Pathway

The findings suggest that health literacy is a key factor influencing patients′ ability to navigate the healthcare system and engage with occupational therapy services. Participants demonstrated limited understanding of healthcare structures, referral processes and professional roles, which may reduce their capacity to actively participate in healthcare decision‐making. These findings align with Smythe et al. [14] who found that patients′ knowledge of healthcare services influences service utilisation and access. Similarly, Charumbira et al. [15] and Naidoo et al. [16] emphasise the importance of patient education in promoting engagement with healthcare pathways.

Poor understanding of referral procedures may also contribute to limited engagement with referral documentation. This finding suggests that patients are not always adequately informed about the purpose of referrals, the nature of occupational therapy services or the expected outcomes of treatment. As a result, patients may assume a passive role within the referral process rather than functioning as active partners in their care. Murphy et al. [17] similarly reported that patients often desire more information regarding referrals, treatment options and recovery expectations. Participants′ uncertainty regarding occupational therapy roles further highlights the need for improved communication during referral. A lack of understanding of the profession may affect expectations of treatment and reduce awareness of the potential benefits of rehabilitation services. Improving patient education at the point of referral may therefore strengthen engagement with occupational therapy and support informed participation throughout the rehabilitation process. Although participants identified concerns regarding service delivery and professional conduct, these experiences appeared less influential than limited healthcare knowledge. Nevertheless, negative interactions with healthcare providers may undermine trust in services and affect healthcare‐seeking behaviour. Delays in emergency care and negative interactions with healthcare providers may also affect patients′ progression through referral pathways by delaying access to subsequent rehabilitation services, including occupational therapy. Patient‐centred communication and respectful professional conduct remain important components of quality care and should be considered throughout referral pathways.

### 4.2. Theme 2: Barriers to Accessing Occupational Therapy Services

The findings indicate that access to occupational therapy is influenced by broader environmental and social determinants of health. Transportation difficulties, long travel distances, adverse weather conditions and concerns regarding community safety collectively affected participants′ ability to attend appointments. These barriers often interacted with one another, increasing the burden associated with accessing healthcare services. Similar findings were reported by Mji et al. [18] who identified transport accessibility, distance, crime and weather as interrelated barriers to healthcare access. The findings further reinforce the importance of considering contextual factors when planning referral pathways. For many patients, attendance at appointments depends on the availability of affordable and reliable transport. Odufuwa [19] similarly identified transportation as a significant challenge affecting patient access to healthcare services. These findings suggest that geographical accessibility should be considered when determining referral destinations, particularly within resource‐constrained settings.

Communication emerged as another important determinant of successful referral. Participants′ accounts suggest that inadequate explanations regarding referral purposes and the use of complex terminology may reduce patients′ understanding of occupational therapy services. Effective communication supports informed decision‐making, strengthens patient engagement and promotes adherence to treatment recommendations. This finding is consistent with Murphy et al. [17], who highlighted the importance of clear and accessible communication throughout the treatment and referral process. Administrative barriers also influenced access to occupational therapy. Although delays associated with referral processing and paper‐based record systems were not universal experiences, they nevertheless contributed to reduced efficiency within referral pathways. Paper‐based documentation continues to be widely used within the South African public health sector [20], making some challenges unavoidable. However, the findings of Day et al. [21] suggest that inconsistent referral procedures and documentation systems may further contribute to delays in service delivery. Improving coordination and administrative efficiency may therefore reduce waiting times and facilitate timely access to rehabilitation services.

### 4.3. Theme 3: Facilitators of Access and Positive Rehabilitation Experiences

In contrast to the barriers identified in the previous themes, Theme 3 highlights factors that supported engagement with occupational therapy services. Timely referrals, collaborative healthcare practices, clear communication and therapist expertise appeared to contribute to positive patient experiences and satisfaction with rehabilitation services. These findings are consistent with those of Rezaie and Kendi [22] and Maseko et al. [23] who identified professional competence, efficient service delivery and coordinated care as important contributors to positive rehabilitation outcomes. Participants′ willingness to prioritise appointments despite environmental and logistical challenges suggests a strong perceived value of rehabilitation services. Motivation to attend appointments appeared to be linked to expectations of improved health and functional recovery. This finding highlights the importance of ensuring that patients understand the purpose and potential benefits of occupational therapy, as positive expectations may enhance adherence to treatment. Although only one participant explicitly referred to negative community perceptions of occupational therapy, this may reflect limited public awareness of the profession and its role in rehabilitation. Misconceptions about occupational therapy could reduce willingness to engage with services and highlight the importance of community education regarding the benefits of rehabilitation. Positive perceptions of occupational therapy were further reinforced by improvements across several ICF domains. Participants described improvements in body functions, including pain management and upper limb function; improved activity performance through enhanced self‐management skills; and increased understanding of assistive technology, which supported participation in everyday occupations and social roles. These findings are consistent with the ICF framework, which conceptualises rehabilitation outcomes as interactions between body functions and structures, activities, participation and contextual factors. The benefits described by participants therefore reflect improvements across multiple dimensions of functioning rather than solely the reduction of physical impairment. Similar outcomes were reported by Maseko et al. [23] where service users described increased participation in meaningful activities following rehabilitation.

The accessibility of referred facilities also appeared to influence adherence. Participants valued facilities that were conveniently located, familiar and easy to reach. These findings support those of Frost et al. [24] who reported that patients prefer healthcare services delivered closer to home. Rezaie and Kendi [22] likewise identified proximity to rehabilitation services as an important facilitator of treatment adherence. The findings therefore emphasise the importance of geographic accessibility when making referral decisions.

Finally, positive perceptions of therapist competence, communication skills and professional conduct contributed substantially to overall satisfaction with services. Participants′ accounts suggest that knowledgeable and supportive therapists increase confidence in the rehabilitation process and enhance patients′ experiences of care. These findings reinforce the importance of maintaining clinical expertise while delivering client‐centred communication that actively involves patients in decision‐making and rehabilitation planning [23].

Overall, the findings demonstrate that successful access to occupational therapy depends on more than the existence of referral pathways alone. Patients must be able to understand, navigate and physically access services for referrals to be effective. Limited health literacy, inadequate communication, environmental barriers and administrative delays may restrict access to occupational therapy, whereas clear communication, collaborative care, therapist expertise and geographically accessible services facilitate engagement with rehabilitation. These findings support the need for patient‐centred referral pathways that promote understanding, accessibility and continuity of care throughout the rehabilitation process.

### 4.4. Contributions and Implications to Practice

• Improve patient education regarding referral processes, occupational therapy services and rehabilitation expectations.
• Strengthen communication between healthcare providers and patients throughout the referral pathway.
• Consider environmental barriers, including transport, distance, weather and community safety, when making referrals.
• Improve interprofessional collaboration and coordination to support continuity of care.
• Reduce administrative delays associated with referral processes and paper‐based systems.
• Promote geographically accessible occupational therapy services to support attendance and treatment adherence.

## 5. Limitations

Several limitations should be considered when interpreting the findings. First, only three healthcare facilities within the Tygerberg substructure were included, limiting the extent to which the findings may represent the experiences of all patients accessing occupational therapy services within the substructure. Poor internet connectivity and background noise occasionally affected the quality of interview recordings, resulting in some inaudible data and the exclusion of one participant′s interview. In addition, the researchers relied on participants′ recollection of events, as access to clinical records was not available, which may have affected the accuracy of information related to referral timelines. Most interviews were conducted in Afrikaans or isiXhosa and translated into English, creating the possibility that subtle meanings were lost during translation. Finally, member checking could not be completed as planned because many participants did not have access to reliable telephonic or electronic communication. Despite these limitations, the study provides valuable insight into patients′ experiences of occupational therapy referral pathways within the Tygerberg substructure.

## 6. Conclusion

The referral process is a vital component of accessing occupational therapy services within South Africa′s public healthcare sector. Several factors were found to facilitate positive referral experiences, including clear communication regarding referrals, consideration of patients′ circumstances when selecting referral facilities, timely referrals and appointments, clear directions to services and positive perceptions of occupational therapy interventions and outcomes.

However, participants also identified barriers that may limit access to occupational therapy services. These barriers related to environmental factors (transport difficulties, distance, adverse weather conditions and community violence), patient‐related factors (limited knowledge of referral processes and occupational therapy services) and health system factors (administrative delays and long waiting times). Consideration of these barriers in future service planning and healthcare policy development may contribute to more patient‐centred, accessible and effective referral pathways, thereby improving rehabilitation outcomes for individuals with upper limb injuries and conditions.

## Funding

This study was supported by Stellenbosch University′s Undergraduate and Honours Research Project Fund.

## Conflicts of Interest

The authors declare no conflicts of interest.

## Data Availability

The data that support the findings of this study are available on request from the corresponding author. The data are not publicly available due to privacy or ethical restrictions.
